# Prognostic role of neoplastic markers in Takotsubo syndrome

**DOI:** 10.1038/s41598-021-95990-9

**Published:** 2021-08-16

**Authors:** Francesco Santoro, Tecla Zimotti, Adriana Mallardi, Alessandra Leopizzi, Enrica Vitale, Nicola Tarantino, Armando Ferraretti, Antonio Giovanni Solimando, Vito Racanelli, Massimo Iacoviello, Michele Cannone, Matteo Di Biase, Natale Daniele Brunetti

**Affiliations:** 1 Department of Medical and Surgical Sciences, University of Foggia, Foggia, Italy; 2 Department of Cardiology, Bonomo Hospital, Andria, Italy; 3 Department of Medicine, Montefiore Medical Center, Cardiology Division, Bronx, NY USA; 4 Department of Cardiology, Caduti Di Guerra Hospital, Canosa, Italy; 5 Department of Biomedical Sciences and Human Oncology, Unit of Internal Medicine “Baccelli”, University of Bari, Bari, Italy; 6 IRCCS Istituto Tumori “Giovanni Paolo II”, Bari, Italy

**Keywords:** Cardiology, Cardiovascular biology

## Abstract

Takotsubo syndrome (TTS) is an acute heart failure syndrome with significant rates of in and out-of-hospital mayor cardiac adverse events (MACE). To evaluate the possible role of neoplastic biomarkers [CA-15.3, CA-19.9 and Carcinoembryonic Antigen (CEA)] as prognostic marker at short- and long-term follow-up in subjects with TTS. Ninety consecutive subjects with TTS were enrolled and followed for a median of 3 years. Circulating levels of CA-15.3, CA-19.9 and CEA were evaluated at admission, after 72 h and at discharge. Incidence of MACE during hospitalization and follow-up were recorded. Forty-three (46%) patients experienced MACE during hospitalization. These patients had increased admission levels of CEA (4.3 ± 6.2 vs. 2.2 ± 1.5 ng/mL, *p* = 0.03). CEA levels were higher in subjects with in-hospital MACE. At long term follow-up, CEA and CA-19.9 levels were associated with increased risk of death (log rank *p* < 0.01, HR = 5.3, 95% CI 1.9–14.8, HR = 7.8 95% CI 2.4–25.1, respectively, *p* < 0.01). At multivariable analysis levels higher than median of CEA, CA-19.9 or both were independent predictors of death at long term (Log-Rank *p* < 0.01). Having both CEA and CA-19.9 levels above median (> 2 ng/mL, > 8 UI/mL respectively) was associated with an increased risk of mortality of 11.8 (95% CI 2.6–52.5, *p* = 0.001) at follow up. Increased CEA and CA-19.9 serum levels are associated with higher risk of death at long-term follow up in patients with TTS. CEA serum levels are correlated with in-hospital MACE.

## Introduction

Takotsubo Syndrome (TTS) is a form of acute heart failure featured by reversible left ventricular dysfunction that can mimic acute myocardial infarction^1^. Several algorithms based on the use of the electrocardiogram and/or biomarkers have been proposed for the diagnostic workup of TTS^1^, but few data are available for long-term prognosis and risk stratification.

TTS is featured by a chronic inflammation process, indeed during the acute phase increased levels of anti-inflammatory interleukins can be found^2^. Moreover, also increased ratio of neutrophil/lymphocyte ratio has been found at admission and is a predictor of in-hospital complication in TTS patients^3^.

Neoplastic markers may represent a surrogate of systemic inflammation and have been evaluated in the context of acute and chronic heart failure^4^. We previously validated the prognostic role of a neoplastic marker, carbohydrate antigen (CA)-125, in the context of TTS^5^. However, there are no data concerning other neoplastic markers and their potential prognostic role in TTS.

Aim of this study was therefore to assess the potential role of neoplastic biomarkers such as CA-15.3, CA-19.9 and Carcinoembryonic Antigen (CEA) in short and long-term prognosis in patients with TTS.

## Methods

### Study population

We prospectively evaluated 90 consecutive patients admitted with TTS to the Department of Cardiology, Ospedali Riuniti, University Hospital of Foggia, Italy, from September 2011 to August 2017 (supplement Fig. S1).

### Inclusion criteria

All patients with suspected TTS underwent coronary and left ventricular (LV) angiography. The diagnosis of TTS was based on the statement of the European Heart Failure Association: (a) Transient regional wall motion abnormalities of LV or right ventricular (RV) myocardium which are frequently, but not always, preceded by a stressful trigger (emotional or physical). (b) The regional wall motion abnormalities usually extend beyond a single epicardial vascular distribution. (c) The absence of culprit atherosclerotic coronary artery disease including acute plaque rupture, thrombus formation, and coronary dissection. (d) New and reversible electrocardiography (ECG) abnormalities (ST‐segment elevation, ST depression, left bundle branch block (LBBB), T‐wave inversion, and/or QTc prolongation) during the acute phase (3 months). (e) Significantly elevated serum natriuretic peptide (BNP or NT‐proBNP) during the acute phase and relatively small elevation in cardiac troponin if compared with the amount of dysfunctional myocardium present. (f) Recovery of ventricular systolic function on cardiac imaging at follow‐up (3–6 months)^6^.

### Exclusion criteria

Patients with history of an active cancer, autoimmune disease, chronic liver disease and pancreatitis were excluded. Active cancer was defined as a cancer diagnosed within the previous 6 months, recurrent, regionally advanced or metastatic cancer, cancer for which treatment had been administered within six months, or hematological cancer that is not in complete remission^7^.

### Clinical and echocardiographic examination

All patients underwent clinical examination; age, gender, medical history, kind of stressors, cardiovascular risk factors as defined by European guidelines^8^ and ECG presentation were recorded. A two-dimensional Doppler echocardiographic examination, on the day of admission, at the third day and at discharge was performed. Two-dimensional echocardiography was performed with commercially available equipment (CX 50 Philips, Best, The Netherlands and VIVID E, GE, Wauwatosa, WI, U.S.A.). Measured variables included right and left ventricular (LV) regional kinesis, using two-dimensionally guided M-mode echocardiography and mitral insufficiency. The LV ejection fraction (LVEF) was calculated using the Simpson method from the apical four-chamber and two-chamber view^9^. Reversible significant MR was defined as reversible moderate to severe MR detected during the first echocardiographic evaluation that disappeared at follow-up examination^10^.

### Blood sample collection

Circulating levels of NT-proBNP, C-reactive protein (CRP), erythrocyte sedimentation rate (ERS), troponin-I (TnI), neoplastic markers (CA-15.3, CA-19.9 and CEA) and interleukins (IL-1a, IL-2, IL-6, IL-10) were obtained by venipuncture at the admission, after 72 h and at discharge. Normal values were < 125 pg/mL for NT-proBNP, < 5 mg/L for CRP, < 11 mm/h for ERS, < 0.5 ng/mL for Troponin I, < 35 UI/mL for CA-15.3, < 40 UI/mL for CA-19.9, 0.1–5.0 ng/mL for CEA, 0.5–1.6 pg/mL for Il-1b, 4.8–8.7 U/mL for IL-2, 1.20–1.95 pg/mL for IL-6 and 0.1–1.8 pg/mL for IL-10.

### Follow-up and definition of outcome

Complete follow-up data were available in all 90 patients with a mean follow-up of 701 days from the time of study inclusion. Patients were scheduled for clinical and echocardiographic examinations at the cardiomyopathy ambulatory of the cardiology department (3 months after TTS episode and every 9 months).

The primary clinical end point was in-hospital major cardiac adverse events (MACE) including death, pulmonary oedema, need for invasive mechanical ventilation, cardiogenic shock, stroke and LV thrombosis. Pulmonary oedema was considered to be present in cases of respiratory distress and pulmonary rales due to pulmonary congestion, as confirmed by chest radiography, a respiratory rate of more than 20 breaths per minute, and an arterial hydrogen ion concentration greater than 45 nmol/L (pH, < 7.35)^11^. Cardiogenic shock was considered to be present if a patient had a systolic blood pressure lower than 90 mm Hg for more than 30 min and clinical signs of pulmonary congestion and impaired organ perfusion, defined as at least 1 of the following: (1) altered mental status; (2) cold, clammy skin and extremities; (3) oliguria (urine output ≤ 30 mL per hour); or (4) arterial lactate level of 2 mmol/L or more (to convert to mg/dL, divide by 0.111)^12^.

At long-term follow-up clinical end points included total mortality, cardiovascular mortality (sudden and non-sudden cardiovascular death) and non-cardiovascular. These clinical end points were recorded and evaluated as adverse events at follow-up. The local ethics committee (Area 1 Foggia—BAT) approved this study, that was consistent with the guidelines of Helsinki. All methods were performed in accordance with the relevant guidelines and regulations. All patients gave a written informed consent.

### Statistical analysis

Continuous variables were reported as means ± standard deviation and compared with Student’s t-test for either paired or unpaired groups as required, dichotomic variables as percentage and compared with χ^2^ test of Fisher test as required. Correlations were analyzed with Pearson’s test. Repeated measures were analyzed with analysis of variance test (ANOVA). Survival rate was reported on Kaplan–Meier plot and analyzed with Log-Rank test and multiple stepwise Cox analysis; hazard ratio (HR) with 95% confidence intervals (CI) were calculated. Variables statistically significant at univariable analysis were included in a multivariable model. Logistic regression was used to identify predictors of in-hospital MACEs; odds ratio (OR) with 95% confidence intervals (CI) were calculated. Receiver operating characteristic curves were reported and compared with Hanley and McNeil method. A *p* value < 0.05 was considered as statistically significant.

## Results

### Patient characteristics

The present analysis is based on 90 patients with confirmed TTS. Mean age was 75 ± 12 years and 10% were male. Mean hospital stay was 8 ± 4 days. LVEF at admission was 35 ± 8% while at discharge EF was 49 ± 6%. Baseline features are reported in Table 1. CEA, CA-15.3, and CA-19.9 levels did not significantly change during hospital stay (ANOVA *p* n.s.).

**Table 1:** Baseline features of patients with versus without events.

	General population	Pts with in-hospital MACE	Pts without in-hospital MACE	*p*
Age	75 ± 1.8	78 ± 9.8	72 ± 13	0.02
Male gender	10%	11%	8%	0.60
Hypertension	77%	73%	78%	0.59
Dyslipidemia	50%	47%	51%	0.75
Obesity	28%	24%	32%	0.40
Smoke habit	14%	17%	13%	0.60
Diabetes	21%	24%	17%	0.43
COPD	30%	33%	29%	0.66
CKD	16%	19%	14%	0.58
Hospitalization days	7.8 ± 3.7	8.7 ± 4.6	7.3 ± 2.6	0.08
History of cancer	13%	7%	17%	0.16
**Laboratory data**				
Admission troponin I (ng/mL)	3.9 ± 5	2.9 ± 3	4.8 ± 7.1	0.09
Admission CRP (mg/L)	68 ± 63	42 ± 59	65 ± 71	0.11
Admission NTproBNP (pg/mL)	16﻿,302 ± 15,591	19,821 ± 16,377	12,975 ± 14,619	0.05
**Echocardiografic features**				
LVEF at admission	35% ± 8%	32% ± 8%	38% ± 8%	0.01
LVEF at discharge	49% ± 6%	49% ± 6%	49% ± 6%	0.81
Reversible mitral insufficiency	18%	23%	14%	0.29
RV involvement	6%	9%	2%	0.18
**ECG features at admission**				
Negative T waves	49%	46%	53%	0.52
ST elevation	47%	53%	42%	0.30
ST depression	1%	2%	0%	0.28

*CKD* chronic kidney disease, *COPD* chronic obstructive pulmonary disease, *ERS* erythrocyte sedimentation rate, *CRP* C-reactive protein, *LVEF* left ventricular ejection fraction, *MACE* mayor cardiac adverse event, *RV* right ventricular.

### In-hospital MACE

**Figure 1:**
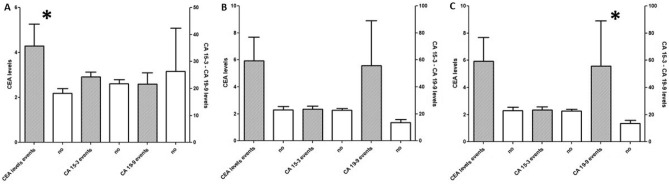
Serum levels of CEA, CA15-3 and CA-19-9 during hospitalization according to incidence of adverse events during hospitalization. *p < 0.05.

Forty-three (46%) patients experienced MACE during hospitalization. Patients experienced the following complications: pulmonary edema (13%, 12 pts), cardiogenic shock (15%, 14 pts), death (12%, 11 pts), stroke (2.2%, 2 pts) and LV thrombi (6.6%, 6 pts). These patients were older (78 ± 9 vs. 72 ± 12 *p* = 0.01), had lower LVEF at admission (32 ± 7vs. 38 ± 8 *p* = 0.01) and higher prevalence of physical stressor (54% vs. 34%; *p* = 0.04) (Table 1). When evaluating serum biomarkers, patients with in-hospital MACE had admission increased levels of NTproBNP (19,821 ± 16,377 vs. 12,975 ± 14,619 pg/mL, *p* = 0.050) and CEA (4.3 ± 6.2 vs. 2.2 ± 1.5 ng/mL, *p* = 0.03) (Table 2, Fig. 1). At discharge CA-19.9 levels were statistically higher among pts with in hospital events (31.2 ± 55.1 vs. 9.1 ± 9.8 UI/mL, *p* = 0.04).

**Table 2:** Laboratory parameters of patients with events versus without events during hospitalization.

	Admission			72 h			Discharge		
	In-hospital MACE	No events	*p*	In-hosp. MACE	No events	*p*	In-hosp MACE	No events	*p*
Troponin I (ng/mL)	4.78 ± 7.65	3.03 ± 2.78	0.14	2.75 ± 6.49	1.46 ± 1.85	0.19	0.28 ± 0.43	0.18 ± 0.23	0.21
CRP (mg⁄L)	64.34 ± 42.29	72.2 ± 58.1	0.13	71.08 ± 41.12	86.2 ± 53.4	0.11	38.01 ± 39.1	14.92 ± 18.13	0.05
NT-BNP (pg/mL)	19,821.5 ± 16,377	12,975.7 ± 14,619	0.05	13,660 ± 11,923	13,274 ± 17,006	0.92	14,333 ± 19,198.4	5653 ± 8474	0.03
**Neoplastic markers**									
CEA (ng/mL)	4.29 ± 6.25	2.18 ± 1.51	0.03	4.36 ± 6.30	5.74 ± 19.73	0.72	4.62 ± 7.76	1.71 ± 1.39	0.05
CA 15–3 (UI/mL)	24.35 ± 11.11	21.77 ± 10.51	0.26	22.80 ± 10.86	20.08 ± 10.19	0.32	22.87 ± 11.57	19.70 ± 9.51	0.24
CA-19–9 (UI/mL)	21.61 ± 27.4	26.32 ± 108.8	0.78	28.58 ± 43.45	38.52 ± 147.07	0.72	31.16 ± 55.06	9.98 ± 9.82	0.04
**Interleukins**									
IL1 ALFA (pg/mL)	0.60 ± 0.26	0.89 ± 0.91	0.11	0.96 ± 1.6	0.83 ± 0.83	0.74	0.76 ± 0.48	1.06 ± 1.7	0.48
IL 2 (UI/mL)	2.61 ± 2.99	3.44 ± 7	0.52	2.80 ± 2.9	3.59 ± 4.2	0.44	4.91 ± 10.95	5.18 ± 5.31	0.91
IL 6 (pg/mL)	72.10 ± 199.8	24.64 ± 58.5	0.14	29.82 ± 39.12	23.02 ± 53.74	0.58	25.02 ± 31.14	7.94 ± 8.05	0.05
IL 10 (pg/mL)	10.38 ± 35.89	2.7 ± 3.34	0.17	2.07 ± 1.99	2.33 ± 2.74	0.68	2.56 ± 3.12	2.30 ± 2.99	0.74

*MACE* mayor cardiac adverse event.

Admission CEA levels above median (> 2 ng/mL) were associated with higher rates of in-hospital MACE (OR 2.7, 95% CI 1.1–6.3, *p* = 0.03), but just at univariate analysis. CEA and CA-19.9 levels at admission were correlated with CRP and NT-proBNP levels (*p* < 0.05, supplement Fig. S2).

### Long term follow-up

During follow-up the incidence of adverse events was 40% (27% death, 24% cardiovascular rehospitalisation, 4% TTS recurrence). Patients were taking the following drug therapy at follow-up: aspirin (75%), betablocker (57%), ace inhibitors/ARB (62%) and statins (72%).

**Figure 2:**
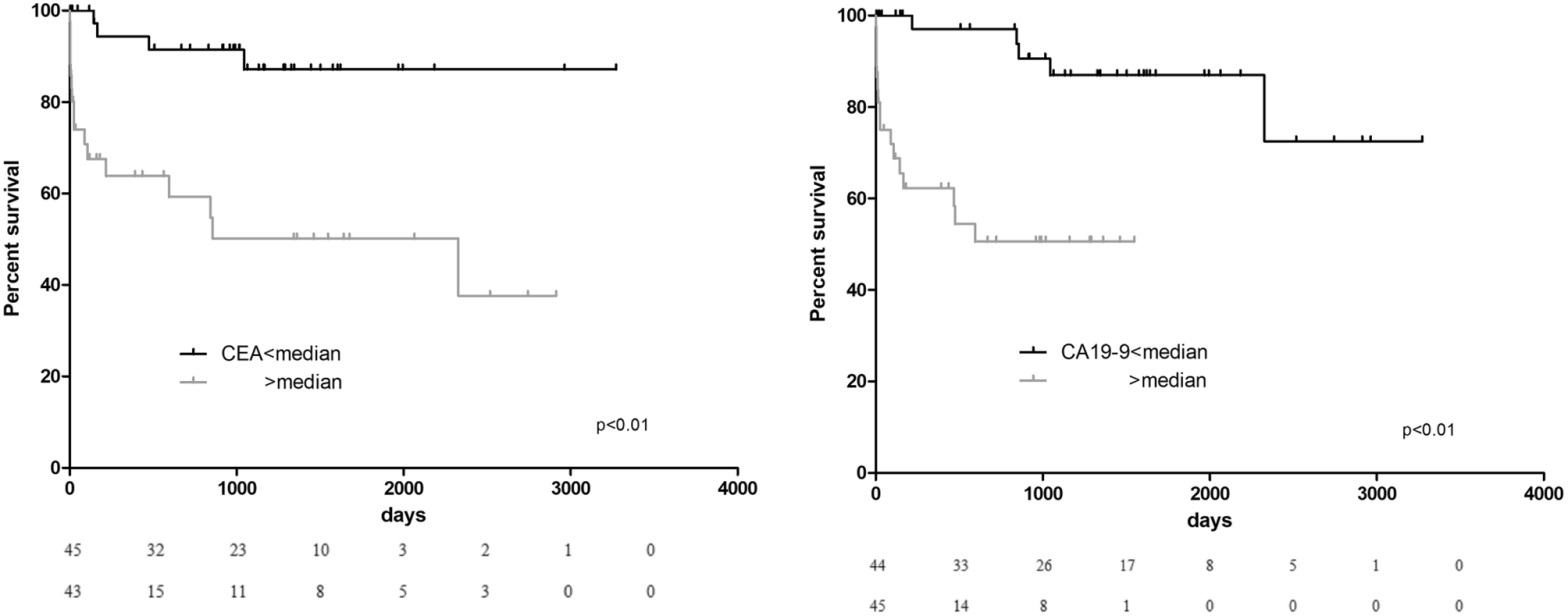
Kaplan Meier curves, showing survival rates according to CEA and CA-19.9 over median values.

**Figure 3:**
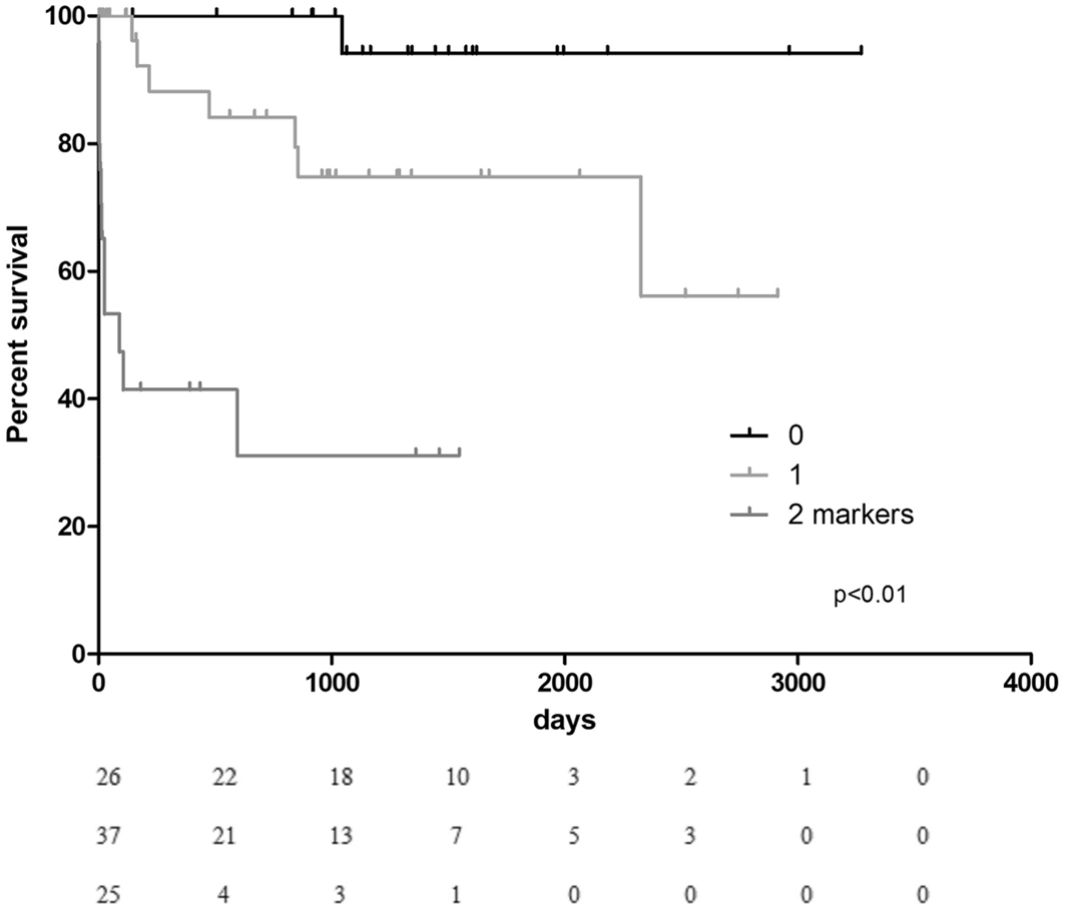
Kaplan Meier curves, showing survival rates in patients with levels of CEA and CA-19.9 over median values or both.

CEA and CA-19.9 levels higher than median (> 2 ng/mL, > 8 UI/mL respectively) were associated with an increased risk of death (Log Rank *p* < 0.01 for both, HR = 5.3, 95% CI 1.9–14.8; HR = 7.8 95% CI 2.4–25.1, respectively, *p* < 0.01) (Fig. 2). Levels higher than median of CEA, CA-19.9 or both were independent predictors of death at long-term follow up, even at multivariable analysis (Log Rank *p* < 0.01). Having both CEA and CA-19.9 levels above median was associated with an increased risk of mortality of 11.8 (95% CI 2.6–52.5, *p* = 0.001) at follow up (Tables 2 and 3, Fig. 3).

**Table 3:** Univariable and multivariable analysis, predictors of mortality at long-term follow up.

	Univariable				Multivariable			
	Hazard ratio	Lower	Upper 95% CI	*p*	Hazard ratio	Lower	Upper 95% CI	*p*
Age	1.15	1.08	1.22	< 0.001	1.15	1.05	1.25	0.0025
Male gender	1.43	0.42	4.85	0.5647				
Hypertension	0.60	0.25	1.47	0.2675				
Dyslipidemia	0.75	0.33	1.71	0.4941				
Obesity	0.98	0.38	2.52	0.9704				
Smoke habit	0.39	0.09	1.66	0.2001				
Diabetes	1.59	0.62	4.08	0.3331				
COPD	1.72	0.75	3.97	0.1997				
CKD	1.57	0.64	3.82	0.3218				
History of cancer	1.09	0.32	3.69	0.8936				
Admission Troponin I (ng/mL)	1.04	0.99	1.09	0.1510				
Admission CRP (mg/L)	1.01	1.00	1.01	0.0118	1.00	0.99	1.00	0.2367
Admission NTproBNP (pg/mL)	1.00	1.00	1.00	< 0.001	1.00	1.00	1.00	0.0271
Admission LVEF	0.00	0.00	0.83	0.0432	0.01	0.00	34.91	0.2878
Reversible Mitral insufficiency	1.34	0.72	2.50	0.3539				
RV involvement	3.68	1.23	11.00	0.0197	1.87	0.57	6.11	0.3021
Negative T waves	0.69	0.30	1.59	0.3865				
ST elevation	1.13	0.50	2.57	0.7637				
ST depression	0.03	0.00	> 100	0.7148				
Number of markers (CEA, CA-19.9) with levels above median	6.15	2.91	13.01	< 0.001	3.92	1.68	9.16	0.0016

*COPD* chronic obstructive pulmonary disease, *CKD* chronic kidney disease, *CRP* C-reactive protein, *LVEF* left ventricular ejection fraction, *RV* right ventricular.

At ROC curve analysis for the prediction of mortality at follow up, area under curve with CEA at admission was larger (79%, 95% CI 69–88%) than with CA-19.9 (70%, 95% CI 58–79%), both CEA and CA-19.9. higher than media (77%, 95% CI 67–86%) and LVEF (64%, 95% CI 53–75%), although the differences are not statistically significant.

## Discussion

We report one of the first studies that evaluate the potential prognostic role of neoplastic markers among patients with TTS. We found that:

1. Increased levels of CEA at admission were associated with higher risk of in-hospital complications;
2. CEA and CA-19.9 levels (higher than median) were associated with increased risk of death at long-term follow-up.
3. At multivariable analysis including age, sex and admission LVEF, CEA and CA-19.9 levels higher than median were an independent predictor of death at long term.

TTS is an acute heart failure syndrome, whose physio-pathology is still unknown. Some authors reported increased serum catecholamine levels as the main driver of reversible myocardial dysfunction^13^ however local adrenal myocyte release may play an important role^14^.

Although it was previously considered a benign disease, recent literature found that TTS is featured by high rate of in-hospital complications^15^ and an annual rate of cardiovascular events of 9.9%^16^. Therefore, according to these data, aim of the study was to evaluate the potential prognostic role of neoplastic markers during hospitalization and at follow-up among TTS patients.

Neoplastic markers in the context of acute and chronic heart failure have been extensively evaluated^17^. Most of the studies focused on CA-125, a glyco-protein normally produced by peritoneal, pleural and pericardial cells whose levels are increased in women affected by ovarian carcinoma, especially with peritoneal involvement^18,19^. CA-125 has proven to be related with congestive heart failure severity and short-term prognosis^20^. Additionally, CA125 levels combined with BNP levels, provide an additional prognostic value and enables better 6-month risk stratification^21,22^. When compared with other neoplastic markers (Alpha-Fetoprotein (AFP), CEA, CA-19.9, CA-15.3), CA 125 seems to be the only one related to the presence and severity of congestive heart failure^23^. We previously found, in a cohort of 63 consecutive TTS patients, that increased CA-125 admission levels are associated with longer hospital stay, lower LVEF and higher risk of adverse events during follow up^5^.

In the present study we found among 90 consecutive TTS patients that Carcinoembryonic antigen (CEA) and CA-19.9 were both associated with higher risk of long-term death, while CEA levels at admission were statistically correlated with in-hospital MACE.

Interestingly, 23 out of 36 (63%) adverse events happened during the first year, mainly death (17 pts) and cardiovascular rehospitalization (6 pts). Therefore, the prognostic role of neoplastic markers is in line with the long-term follow-up of TTS patients (Figs. 2 and 3).

Carcinoembryonic antigen (CEA) is a glycoprotein normally produced in gastrointestinal tissue during fetal development and usually present at low levels in healthy adults. However, its serum levels are raised in colon-rectal carcinoma and in heavy smokers^24,25^.

CA-19.9 is a glycoprotein that can be found on the epithelium of the fetal stomach, intestine, liver and pancreas, and traces can be detected in adult gastrointestinal tract and lung tissue. It is mainly used for assessing prognosis and monitoring therapy response of pancreatic and gastro-intestinal cancers^26^.

Combination of CEA and CA-19.9 levels can provide a better risk stratification and detection of recurrence for colon-rectal cancer^27,28^. Recent data showed that also among patients with chronic obstructive pulmonary disease, CEA and CA-19.9 are associated with the severity of the disease^29^.

These data are in line with the finding of the present study in which levels of CEA and CA-19.9 both higher than median were able to better stratify long term mortality.

Increased values of CEA and CA 19.9 in Takotsubo patients may be related to the inflammation of serosal tissue due to catecholamine release and to patient’s comorbidities. Accordingly, in the present study, CEA and CA-19.9 levels at admission were correlated with CRP levels. Moreover, the mayor prognostic factors after TTS episode are non-cardiovascular comorbidities^30^.

Cancer among TTS patients is not uncommon, has an incidence of 15–20%^31^ and is associated with higher risk of in-hospital events (mainly due to higher need for respiratory support) and of adverse events at follow-up^32^. According to current literature, there are no specific subsets of cancer related to TTS; in some cases, TTS could represent a paraneoplastic phenomenon^33^. Therefore, among patients without a clear triggering TTS stressor, cancer screening should be performed. In the present cohort only one patient experienced gastric cancer after TTS and presented at admission with CEA but not CA-19.9 values higher than median.

Moreover, patients with TTS may have a chronic inflammation; during the acute and subacute phase of TTS, higher serum levels of anti-inflammatory interleukins are present in TTS than in acute myocardial infarction^34^ and increased serum admission levels of Interleukin-6 and Interleukin-10 in TTS are associated with higher risk of adverse events during follow-up^35^.

The present study reinforces the concept that TTS is cardiac disease that reflects comorbidities of the patient affected and subclinical chronic inflammation may be present even before admission. Neoplastic markers could be useful for a better risk stratification of TTS patients; however additional research is needed on large multicenter registries.

## Conclusions

Increased CEA and CA-19.9 serum levels are associated with higher risk of death and adverse events at long-term in patients with TTS. CEA serum levels are correlated with in-hospital MACE.

### Limitations

These are preliminary results to be confirmed in larger cohorts of patients. Data concerning clinical frailty of the patients were not prospectively recorded. Further and more adequately-powered prospective studies are warranted to clarify the optimal cut-off, and the prognostic value of all neoplastic markers.

## Supplementary Information

Supplementary Figures.
