# Supplementary material for: Prognostic role of neoplastic markers in Takotsubo syndrome
Source: Sci Rep. 2021 Aug 16;11:16548. doi: 10.1038/s41598-021-95990-9 (PMC8368165; doi:10.1038/s41598-021-95990-9)
Supplement: Supplementary file 1 — Supplementary Figures. [file 41598_2021_95990_MOESM1_ESM.docx]

**Prognostic role of neoplastic markers in Takotsubo syndrome.**

Francesco Santoro, MD, PhD ^1,2^, Tecla Zimotti, MD^1^, Adriana Mallardi, MD^1^, Alessandra Leopizzi, MD^1^, Enrica Vitale, MD ^1^, Nicola Tarantino, MD ^3^, Armando Ferraretti, MD ^4^, Antonio Giovanni Solimando MD, PhD ^5 6^, Vito Racanelli MD, PhD ^5^, Massimo Iacoviello, MD, PhD ^1^, Michele Cannone, MD^2^, Matteo Di Biase MD^1^, Natale Daniele Brunetti, MD, PhD^1^.

^1^ University of Foggia, Department of Medical and Surgical Sciences, Foggia, Italy;

^2^ Department of Cardiology, Bonomo Hospital, Andria, Italy.

3 Department of Medicine, Cardiology Division, Montefiore Medical Center, Bronx, New York, USA.

4 Department of Cardiology, Caduti di Guerra Hospital, Canosa, Italy.

5 Department of Biomedical Sciences and Human Oncology, Unit of Internal Medicine "Baccelli", University of Bari, Italy.

6 IRCCS Istituto Tumori "Giovanni Paolo II" , Bari, Italy


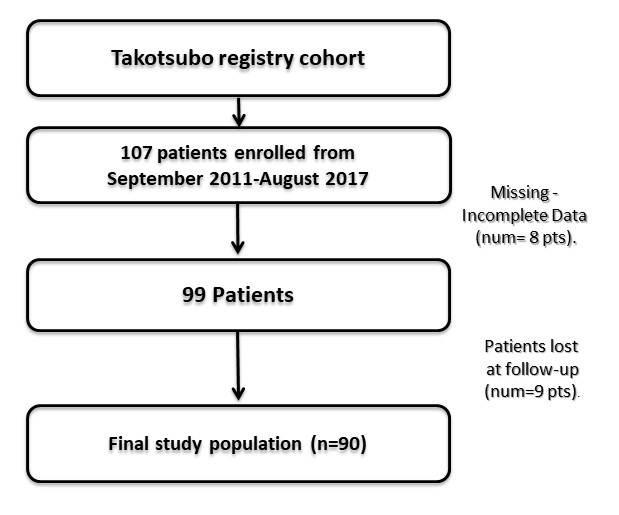


**Supplement Figure 1.** Flow-chart study showing patients included in the study.


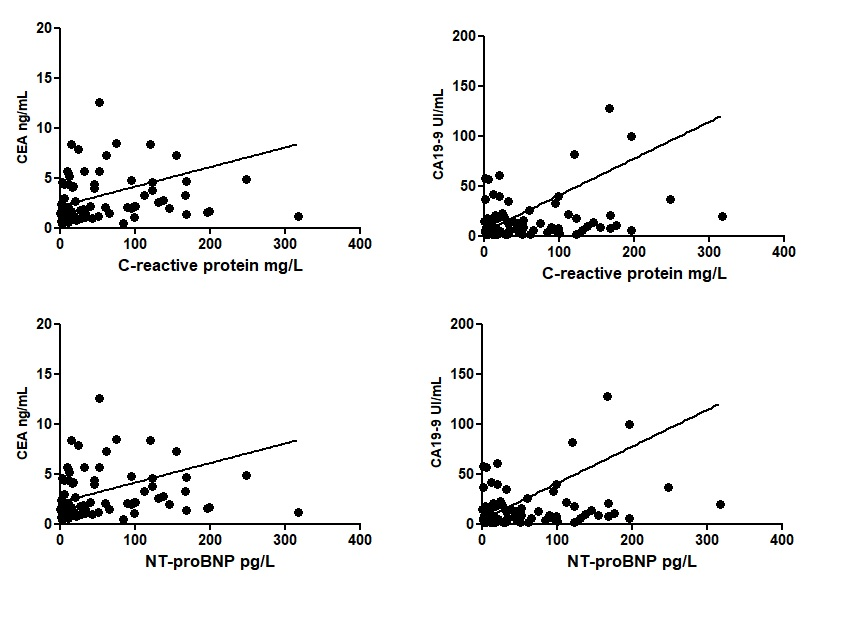


**Supplement Figure 2.** Correlation between admission CEA, CA-19.9 levels and .C-reactive protein, NT-proBNP.
